# miRNA Profiling in the Chicken Liver under the Influence of Early Microbiota Stimulation with Probiotic, Prebiotic, and Synbiotic

**DOI:** 10.3390/genes12050685

**Published:** 2021-05-01

**Authors:** Michalina Sikorska, Maria Siwek, Anna Slawinska, Aleksandra Dunislawska

**Affiliations:** 1 Department of Animal Biotechnology and Genetics, UTP University of Science and Technology, 85-796 Bydgoszcz, Poland; msikorsk@med.uoa.gr (M.S.); siwek@utp.edu.pl (M.S.); slawinska@utp.edu.pl (A.S.); 2 Department of Medicine, National and Kapodistrian University of Athens, 11527 Athens, Greece

**Keywords:** bioactive substances, host-probiotic interaction, in ovo technology, intestinal microbiota

## Abstract

Epigenetic regulation of gene expression is a form of interaction of the external environment on reading and transcription of genetic information encoded in nucleic acids. We provided evidence that early stimulation of the chicken microbiota with in ovo delivered synbiotics influenced gene expression and DNA methylation in the liver. Therefore, we hypothesize that the stimulation of microbiota by administering bioactive substances in ovo also affects the activity of miRNA in liver. For the analysis of miRNA activity, RNA was isolated from liver of adult broiler chicken and native chicken breed. The animals received a prebiotic, probiotic and synbiotic in ovo on day 12 of egg incubation. The analysis of miRNA expression was performed using the LNA method on a miRNA panel selected on the basis of previous microarray experiments. We have found increased miRNA expression activity after probiotic and synbiotic administration, especially in native chicken breed. Our results suggest that prebiotics reduce or do not affect miRNA activity. We have also shown that miRNA activity is regulated by the substance and genotype of the chicken. We can conclude that miRNAs constitute an important component of the molecular mechanism of host–probiotic interaction in liver.

## 1. Introduction

Host-microbiota crosstalk leads to measurable modulation of the molecular pathways [1]. Early stimulation of the chicken microbiota with in ovo delivered synbiotics on day 12 of egg incubation influenced gene expression [2] and DNA methylation [3] in the liver. *Lactobacillus* synbiotic delivered in ovo hypermethylated *ANGPTL4* gene, which is involved in the metabolic pathways related to decreased lipoprotein lipase activity, triglyceride homeostasis, and angiogenesis [3]. Hypermethylation results in the silencing of the *ANGPTL4* gene expression. Another gene which has been epigenetically changed upon synbiotic administration is *NR4A3*, which is responsible for the regulation of fatty acid use, muscle mass, cell proliferation and differentiation and also promotes food intake and body weight gain [3]. Upregulation of the *NR4A3* gene expression is associated with a significant decrease in methylation.

Changes in the DNA methylation levels can be passed on to the next generation [4,5]. By impacting the expression of the metabolism-related genes in the chickens, one can permanently modulate their metabolic traits. There are two significant epigenetic mechanisms which can regulate gene expression: DNA methylation and activity of microRNA (miRNA) particles. DNA methylation is based on the addition of the methyl groups to the CpG islands (regions with a high frequency of CpG sites) of the DNA strand, which block the access of the transcription factors [6]. In turn, miRNAs are small RNA molecules encoded in the genome that impede the expression of the target genes. The mature miRNA binds to the three prime untranslated region (3’-UTRs) end of the mRNA molecule of the regulated target gene, destabilizing it and preventing translation. The effect of this process is target gene silencing. This binding is non-homologous, therefore making single miRNAs capable of regulating hundreds of target genes [7]. There are an increasing number of publications aimed at understanding the complex role of miRNA in epigenetic regulation [8,9]. Epigenetic modulators, such as miRNAs, influence the protein levels of final mRNA without any changes in their gene sequences. It is possible that the combination of the epigenetic pathway with the action of miRNAs results in changes of gene expression by creating a miRNA–epigenetic feedback loop [10]. miRNAs function as the key regulators and determinants in several crucial cellular processes: developmental timing, neuronal cell fate, cell death, fat storage and proliferation [11]. It has been described in a number of studies that embryonic growth corresponds to the diverse changes caused in the various expression patterns of miRNAs through many tissues [12,13].

The in ovo technique is an effective method for an early microbiome programming based on successful colonization of the intestinal microbiome with the commensal microbiota. This method is precise and allows modulation of the conditions inside the egg. Day 12 is used to inject prebiotics, probiotics, and synbiotics, what induce cross-correlations that positively affect the gut microbiota, thereby stimulating the native microbiota [14]. Prebiotics, due to their relatively small size, penetrate the inner and outer membranes of the egg which gives the effect of stimulation of the congenital microbiota in embryonic intestines [14,15]. Contact with the probiotic deposited in the air chamber occurs on day 18 of embryonic development due to the fact that during this period the inner shell membrane is mechanically ruptured by the chick’s beak [14,15,16]. The intestinal microbiota after in ovo stimulation is stable enough to exclude competition and it programs for the life span. The in ovo strategy concerns the reprogramming of embryonic gastrointestinal tract (GIT) colonization with native microbes, which allows the expected effect to be achieved in a short period of time during the egg incubation [14].

Our previous studies proved that the in ovo administration of a bioactive substance at the stage of embryonic development of chickens affects epigenetic mechanism in liver (DNA methylation, which also leads to silencing of gene expression) [3,17]. Therefore, we hypothesize that the stimulation of microbiota by administering bioactive substances in ovo also affects the activity of miRNA in liver. As proven, probiotics, prebiotics and synbiotics affect the expression profile of miRNAs, hence a common control mechanism between the microbiota and the host is evident [18].

The aim of this study was to analyze selected miRNAs in liver that regulate a large number of genes after the administration of a prebiotic, probiotic and synbiotic in ovo in two different chicken genotypes—broiler chicken and native chicken breed. Feed and water were delivered manually and were available ad libitum. The feeding regime was applied according to the requirements of the given genotype.

## 2. Materials and Methods

### 2.1. The Experimental Design

We incubated 600 eggs of Ross 308 (Ross) broiler chicken and 600 eggs of Green-legged Partridgelike (GP; native chicken breed) at a temperature of 37.8 °C and relative humidity between 61% and 63% in a commercial hatchery (Drobex-Agro, Solec Kujawski, Poland). On the day 12 of incubation, eggs were randomly distributed into experimental groups (150 eggs per group): (1) probiotic (PRO)– *Lactococcus lactis* subsp. *cremoris* IBB477; (2) prebiotic (PRE)– galactooligosaccharides (GOS; Bi^2^tos; Clasado Biosciences, Ltd., Jersey, UK); (3) synbiotic (SYN)—*Lactococcus lactis* subsp. *cremoris* with GOS. The set amount of bacteria was 10^5^ bacteria CFU/egg and the amount of prebiotic was 3.5 mg/egg. The control group (C) was mock-injected with 0.2 mM physiological saline (0.9%). Eggs were injected into an air chamber with 0.2 mL of aqueous solution of each substance. After hatching, birds were housed in litter pens (4 replicates/group, 8 animals each). The main experiment was conducted in experimental station at the University of Life Sciences in Wroclaw, Poland.

**Figure 1:**
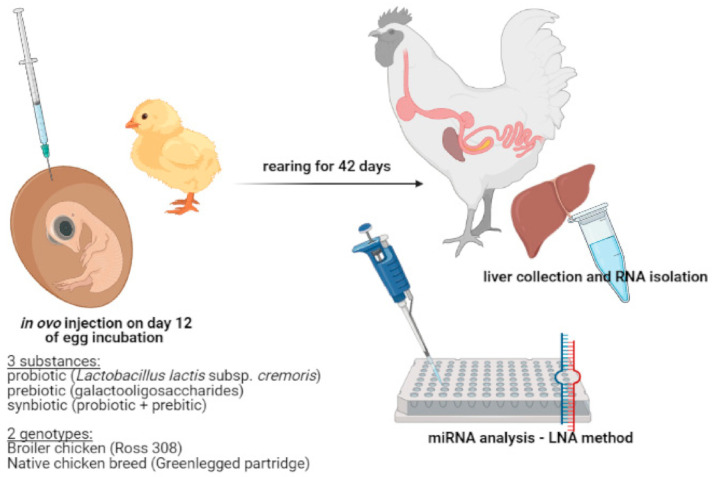
Experimental setup (created with BioRender.com, https://biorender.com/, accessed on 30 April 2021).

Five randomly selected individuals from each group (PRO, PRE, SYN and C) were sacrificed on the day 42 post-hatching and liver was collected. The experiment was approved by the Local Ethics Committee for Animal Experiments (Bydgoszcz, Poland) (study approval reference number 16/2014). All methods were carried out in accordance with relevant guidelines and regulations. Experimental setup is presented in Figure 1.

### 2.2. Isolation of RNA from Liver

Livers for RNA isolation (*n* = 5 per group) were stored in stabilization buffer (fix RNA, EURx, Gdańsk, Poland). RNA isolation was prepared by using TRI reagent (MRC, Cincinnati, OH, USA) and commercial kit for RNA purification (Universal RNA Purification Kit, EURx, Gdańsk, Poland). Liver was homogenized with the TissueRuptor II homogenizer (Qiagen GmbH, Hilden, Germany) in TRI reagent. RNA quality and quantity was checked by electrophoresis (2% agarose gel) and NanoDrop2000 (Scientific Nanodrop Products, Wilmington, NC, USA).

### 2.3. miRNA Selection

MiRNA selection was based on two set of microarray data (Chicken Gene 1.1 ST Array Strip, Affymetrix, Santa Clara, CA, USA) in immune and metabolic tissues. These data sets contained broiler chicken transcripts generated from individuals which received prebiotics and synbiotics in ovo on day 12 of egg incubation [17,19]. Selection of miRNAs for analysis was made by means of quantitative analysis of genes (based on the TargetScan software) (Table 1), which are regulated by the given miRNAs. Subsequent analysis were performed with the miRNAs regulating the highest number of genes.

### 2.4. LNA Method

Analysis of miRNA activity was performed using the miRCURY LNA miRNA PCR Assay (Qiagen, Hilden, Germany) according to the manufacturer’s protocol. Reverse transcription was performed with miRCURY LNA Kit cDNA. Reverse transcription-quantitative polymerase chain reaction (RT-qPCR) was performed in a total volume of 10 μL, which included miRCURY LNA miRNA SYBR Green qPCR Master Mix (QIAGEN, Hilden, Germany), 1 μM of each primer and 3 μL of diluted cDNA (1:60). Primer sequences were derived designed with NCBI Primer Blast, based on two sets of microarray data. Thermal cycling was conducted in LightCycler II 480 (Roche Applied Science, Basel, Switzerland). qPCR thermal profile consisted of PCR initial heat activation denaturation at 95 °C for 2 min, 45 cycles of amplification including 10 s of denaturation at 95 °C, 60 s of combined annealing and extension at 56 °C. After completion of the amplification reaction, a melting curve was generated to test the specificity of RT-qPCR. For this purpose, the temperature was gradually increased from 60 °C to 95 °C with continuous fluorescence measurement. The analysis was performed in 5 biological replications for a given group, and in 2 technical replications for the sample.

PCR Primers (Table 2) were delivered by Qiagen (miRCURY LNA miRNA PCR Assay). Regarding the hsa-miR-204-5p Primer, gga-miR-211 was ordered, but due to the lack of availability of gga-miR-211, based on the same nucleotide sequence, hsa-miR-204-5p was used.

### 2.5. Data Analysis

We used The LightCycler 480 Software, Version 1.5 (Roche Applied Science, Basel, Switzerland) for generating the cycle threshold (Ct) values. Ct is the number of multiplication cycles that are required for the RT-PCR product signal to pass a specific threshold. The Ct value indicates the point at which the threshold cycle was exceeded, which indicates that the lower the Ct value, the higher the miRNA activity. The mean of 5 biological replicates was presented for data visualization. Statistical analysis was performed using the *t*-test (*p* < 0.05), where the study groups were compared with the control group. An interaction analysis was performed for 3 miR where data for both genotypes were generated. The significance of effects: genotype, substance and interaction genotype × substance were calculated with two-way ANOVA (SAS Enterprise Guide 8.2 update 4; SAS Institute Inc., Cary, NC, USA).

## 3. Results

### miRNA Profiling

**Figure 2:**
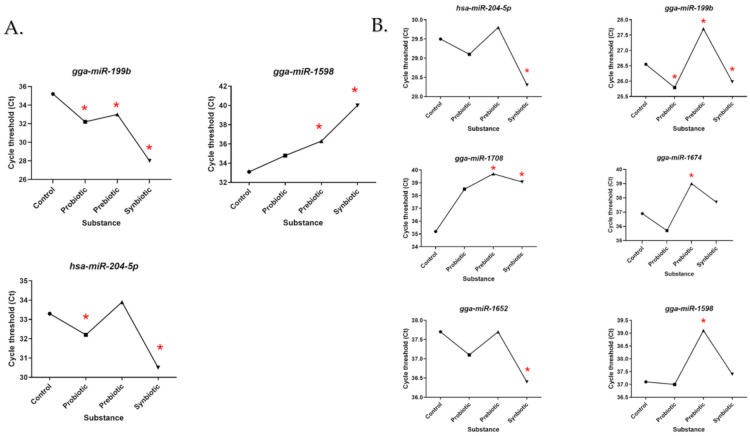
Activity of miRNA in (**A**) broiler chicken (Ross 308) and (**B**) native chicken breed (Green-legged Partridgelike) after in ovo administration of probiotic prebiotic, and synbiotic based on average Ct value. The control is the group that received saline in ovo. The significance of changes in the miRNA activity was analyzed by *t*-test (*p* < 0.05) and labeled with (*).

Based on LNA analysis, profiles of miRNA activity were obtained after administration of individual substances in two chicken genotypes. Figure 2A shows miRNA profiling versus in ovo substance administration, where statistically significant results were obtained in the liver of the chicken broiler. In contrast, Figure 2B shows analytical results for native chicken breed (GP). The Ct value indicates the point at which the threshold cycle was exceeded, which indicates that the lower the Ct value, the higher the miRNA activity. Ross showed an increase in miR-199b activity in the synbiotic group (lower Ct value compared to the others) and low activity in the control group. MiR204-5p shows a similar relationship to miR-199b. For miR-1598 there was a decrease in activity in all groups, especially after administration of the synbiotic. In GP miRNAs—miR204-5p, miR-199b, miR-1674, miR-1652 and miR1598 show the same relationship—an increase was seen in their activity after the administration of a probiotic and a synbiotic (low Ct), while a decrease (high Ct) or no change compared to control was seen after the administration of a prebiotic. The miR-1708 profile shows the lowest activity in the prebiotic group, while the highest was in the control group.

In the case of the other miRNAs, where plots were not presented, no detection or a possible Ct value greater than 40 was shown. Effects and interaction analysis were performed for the three miRNAs where data for both genotypes was obtained. The results are shown in Table 3.

## 4. Discussion

In this study we analyzed miRNA profiles in two distinct chicken genotypes, which were influenced by delivery of a prebiotic, probiotic, and synbiotic in ovo on day 12 of eggs incubation. The experimental design was aimed to support the hypothesis that epigenetic mechanisms regulates gene expression in chickens stimulated in ovo and is specifically involved in metabolic gene expression in the liver.

The liver plays a key role in the metabolism of nutrients such as carbohydrates, proteins and lipids, participates in fat digestion, blood protein synthesis and protein balance is and filters the blood from toxic substances. In addition, the liver performs some of the body’s immune functions [20]. It hosts hepatic macrophages, which are mainly responsible for the production of inflammatory mediators [21]. An important function of the liver is the ability to recruit and activate immune cells in response to metabolic signals from the intestines [22]. In addition, there is an ample evidence of a link between the intestines and the liver due to the connection of the gut via the bile ducts, portal vein, and systemic circulation to the liver. These two organs communicate in a bidirectional manner. The first route of communication between the liver and the intestine is the release of bile acids and mediators into the bile ducts and systemic circulation. On the other hand, the intestinal microbiota is involved in the metabolism of the bile acids and amino acids, but also metabolizes dietary components, which are transported to the liver through the portal vein [23]. Thus, liver is involved in interactions between the intestinal microbiota and the host metabolism [24]. Evidence from our previous research suggests that the silencing of gene expression in the liver after the in ovo administration of synbiotics might have an epigenetic background [17]. It was already confirmed by the analysis of the DNA methylation in the liver [3]. The following step in the deciphering of an epigenetic regulation of gene expression is the analysis of miRNA activity. At this stage we have adopted the study of the effect of the synbiotic, but also of its individual components (prebiotic and probiotic). Analyses were performed based on two genotypes of different selection history and origin. Ross 308 broiler chickens are a selected breeding line which is famous for fast-growing possibilities. They is characterized by an excellent pace of weight gain and production parameters. Green-legged Partridgelike chickens are a common breed of Polish descent. This breed has a rather different application than the Ross broiler chickens due to their low body weight and slim body form and the lack of such intensive selection. It is an important animal model for research due to their considerable strength and ability to adapt easily to new climatic conditions. It is characterized by high disease resistance and low environmental and food requirements [25].

In this study, we showed significant changes in the activity of three out of ten analyzed miRNAs in the liver of broiler chickens and six of native chickens. By analyzing the synbiotic and its individual components—probiotic and prebiotic—we could determine which component of the synbiotic plays an important role in the regulation of miRNA activity. The analyses were based on two different chicken genotypes of a different origin and selection history—broiler chicken (Ross) and native chicken (GP). Ross is a meat-type which was created as a result of intensive genetic selection. GP is characterized by high disease resistance and low environmental and food requirements [25]. The resulting differences can be significantly reflected in epigenetic mechanisms, indicating the differences resulting from the genetic background.

Disturbances in miRNA expression can result in abnormalities in numerous cellular processes. The miRNAs analyzed in this study take part in distinct functions. miR-199b is related to lymphomagenesis, so it may modulate the regulatory pathways in chicken embryo [26]. Glazov et al. [27] found miR-1598 down-regulated in *Epidermodysplasia verruciformis* following treatment with AIV, LPS and polyl:C, which may be related with cell regeneration processes. miR-204-5p is associated with melanogenesis and its expression differs considerably between black and white bulb feathers. It may affect the different colors of skin or feathers in animals [28]. miR-1708 is involved in promoting clathrin-dependent endocytosis, which may cause changes in the absorption processes of the cell [29]. miR-1652 is significantly expressed in chicken PGCs, which may affect the development of the embryo [30]. miR-1674 was associated by Hong et al. with a resistance to necrotizing enterocolitis, which may be crucial in supporting treatments of necrotizing enterocolitis disease [31].

Host miRNAs are able to inhibit or stimulate the growth of specific microorganisms present in the gut [32]. The literature has shown that modulation of the gut environment has a significant effect on the regulation of epigenetic mechanisms in animals. The influence of microbiome–host interactions on the modulation of miRNA profiling was demonstrated by Dalmasso et al. [33]. Germ-carrying mice were colonized by the gut microbiota of the pathogen-free mice. Consecutive microarray miRNA expression profiling showed differential expression of one miRNA in the ileum and eight in the large intestine. In mammals, the large intestine is the part of the gastrointestinal tract most strongly colonized by microorganisms. In addition, there are a number of reasons for the association of miRNA activity with DNA methylation modification through interaction with newly formed mRNA strands of a given target gene. The major methyltransferases in animals, i.e., DNTM1, 3A and 3B, are assumed to be regulated by miRNAs [9]. This has been proven for the DNTM13B gene in chickens, the expression of which is regulated post-transcriptionally via miRNAs: miR-1741, miR-16c, miR-222 and miR-1632 [34]. Kellermayer et al. [35] analyzed the transcripts and epigenome of the colon mucosa knockout mice in the region of the TLR2 gene that is responsible for recognizing Gram (+) bacterial motifs (probiotic and commensal bacteria belong to Gram (+). In our study, we have found increased miRNA expression activity after probiotic and synbiotic administration, especially in native chicken breed (GP). The activity of miRNA after administration of a synbiotic may be due to the probiotic component. miRNAs are an important regulator of the expression of host genes, while some of them are significantly associated with a given microbial community, and even with specific groups of bacteria [36]. Host-derived miRNAs were also shown to be able to enter a bacterial cell to promote or inhibit specific bacteria.

Hence, it can be concluded that miRNAs constitute an important component of the molecular mechanism of host–probiotic interaction, in particular their participation in gene expression silencing and protein synthesis [18]. It has been proven that a probiotic can participate in the interaction between the microbiota and the host and influence miRNA expression [37]. Similarly, Rodriguez-Nogales and his research group reported increased expression of miRNAs responsible for alleviating inflammation after prior administration of a probiotic containing a strain of *Lactobacillus plantarum* [38]. Supporting conclusions were provided by Heydari et al. [39]. The activity of miRNAs associated with colon cancer increased after administration of probiotics containing *Lactobacillus acidophilus* and *Bifidobacterium bifidum* [39]. In addition, probiotics were shown to have a positive effect on the stimulation of the immune system. It was observed that probiotics containing *Lactobacillus rhamnosus GG* decreased the expression of p38 MAP kinase. This component likely regulates immune system responses by increasing the expression levels of miRNAs such as miR-155, and decreasing the expression of miR-146a, which targets NFκB [40]. Research also shows that miRNAs play an important role in the development of the immune system and the regulation of the host’s inflammatory response. The administration of a probiotic can be effective in alleviating inflammation caused by *Salmonella* infection in poultry [41]. In summary, miRNAs are an important mediator of interactions between the host and the gut microbiota. Probiotics can also stimulate intestinal cells to produce miRNAs with key regulatory functions, thereby affecting the host. This information and the results obtained allow us to conclude that miRNAs also participate in the gut–liver axis and their activation is related to probiotics. Our results suggest that prebiotics reduce or do not affect miRNA activity. Research also suggests that the role of the prebiotic alone in miRNA activity is negligible. Its potential lies in being a component of a synbiotic, where it supports the growth of the bacteria and enhances the effect of the probiotic.

We have proved that some miRNAs show stronger activity precisely in the case of combining a probiotic with a prebiotic than the effects of the probiotic itself. The lack of significant differences between the two analyzed genotypes suggests stronger impact of the bioactive substance compared to genetic background. The influence exerted comes from the delivered substance and it is not related with genotype of chicken.

## Figures and Tables

**Table 1:** Quantitative analysis of genes regulated by miRNA data using the TargetScan software (targetscan.org).

miRNA	Number of Regulated Genes
miR1598	464
miR199B	467
miR1580	750
miR1708	565
miR1674	348
miR1739	993
miR211	241
miR1807	1166
miR1652	734
miR1612	1876

**Table 2:** Primers used to LNA reaction (miRCURY LNA miRNA PCR Assay, Qiagen).

Name	Accession in mirBase	Catalog No.
gga-miR-1580	MI0007306	YP02116472
gga-miR-1612	MI0007340	YP02104755
hsa-miR-204-5p	MI0000284	YP00206072
gga-miR-1708	MI0007444	YP02101847
gga-miR-1807	MI0007552	YP02116728
gga-miR-1674	MI0007408	YP02110404
gga-miR-1652	MI0007384	YP02118325
gga-miR-1739	MI0007478	YP02102609
gga-miR-1598	MI0007325	YP02105156
gga-miR-199b	MI0007426	YP02107667

**Table 3:** Effects of genotype and substance delivered in ovo, and their interaction on miRNA activity (Ct values) in liver of Ross and GP chickens. The significance of effects: genotype, substance and interaction genotype × substance were calculated with two-way ANOVA; ns—not significant.

miR	Genotype	Substance	Genotype × Substance
miR-199b	0.0001	0.0002	0.0038
miR-1598	Ns	0.045	0.044
miR-204-5p	0.0001	0.004	Ns

## Data Availability

New data were created, analyzed and presented in this study. Data sharing is not applicable to this article.
